# Multi-level integrative analysis of the roles of lncRNAs and differential mRNAs in the progression of chronic pancreatitis to pancreatic ductal adenocarcinoma

**DOI:** 10.1186/s12864-023-09209-4

**Published:** 2023-03-06

**Authors:** Zhirong Zhao, Qiang Luo, Yi Liu, Kexin Jiang, Lichen Zhou, Ruiwu Dai, Han Wang

**Affiliations:** 1 Affiliated Hospital of Southwest Jiaotong University, The General Hospital of Western Theater Command, Chengdu, 610031 Sichuan China; 2 Department of Cardiology, Affiliated Hospital of Southwest Jiaotong University, The Third People’s Hospital of Chengdu, Chengdu, Sichuan China; 3 School of Medicine, Jianghan University, 430056 Wuhan, Hubei China; 4 Pancreatic injury and repair Key laboratory of Sichuan Province, The General Hospital of Western Theater Command, Chengdu, Sichuan China

**Keywords:** Chronic pancreatitis, Pancreatic ductal adenocarcinoma, lncRNA, WGCNA, ceRNAs

## Abstract

**Background:**

Pancreatic ductal adenocarcinoma (PDAC) is one of the most malignant tumors and approximately 5% of patients with chronic pancreatitis (CP) inevitably develop PDAC. This study aims explore the key gene regulation involved in the progression of CP to PDAC, with a particular emphasis on the function of lncRNAs.

**Results:**

A total of 103 pancreatic tissue samples collected from 11 to 92 patients with CP and PDAC, respectively, were included in this study. After normalizing and logarithmically converting the original data, differentially expressed lncRNAs (DElncRNAs) and mRNAs (DEGs) in each dataset were selected. To determine the main functional pathways of differential mRNAs, we further annotated DEGs using gene ontology (GO) and analyzed the Kyoto Encyclopedia of Genes and Genomes (KEGG) pathway enrichment. In addition, the interaction between lncRNA-miRNA-mRNA was clarified and the protein–protein interaction (PPI) network was constructed to screen for key modules and determine hub genes. Finally, quantitative real-time polymerase chain reaction (qPCR) was used to detect the changes in non-coding RNAs and key mRNAs in the pancreatic tissues of patients with CP and PDAC. In this study, 230 lncRNAs and 17,668 mRNAs were included. There were nine upregulated lncRNAs and 188 downregulated lncRNAs. Furthermore, 2334 upregulated differential mRNAs and 10,341 downregulated differential mRNAs were included in the enrichment analysis. From the KEGG enrichment analysis, cytokine–cytokine receptor interaction, calcium signaling pathway, cAMP signaling pathway, and nicotine addiction exhibited significant differences. Additionally, a total of 52 lncRNAs, 104 miRNAs, and 312 mRNAs were included in the construction of a potential lncRNA-miRNA-mRNA regulatory network. PPI network was established and two of the five central DEGs were created in this module, suggesting that lysophosphatidic acid receptor 1 (LPAR1) and regulator of calcineurin 2 (RCAN2) may play significant roles in the progression from CP to PDAC. Finally, the PCR results suggested that LINC01547/hsa-miR-4694-3p/LPAR1 and LINC00482/hsa-miR-6756-3p/RCAN2 play important roles in the carcinogenesis process of CP.

**Conclusion:**

Two signaling axes critical in the progression of CP to PDAC were screened out. Our findings will be useful for novel insights into the molecular mechanism and potential diagnostic or therapeutic biomarkers for CP and PDAC.

**Supplementary Information:**

The online version contains supplementary material available at 10.1186/s12864-023-09209-4.

## Background

Pancreatic ductal adenocarcinoma (PDAC), one of the most malignant tumors, is characterized by insidious onset and difficulty in early diagnosis [1, 2]. Notably, PDAC-associated morbidity and mortality are increasing rapidly worldwide [3]. The American Cancer Society estimates PDAC to be the second leading cause of cancer-related death in the United States by 2030 [4]. Currently, the primary treatment strategy used for alleviating PDAC is surgery. However, most patients with PDAC are often found to be outside the indications of surgery owing to the lack of specific symptoms and easy early micrometastasis [5, 6]. Therefore, researchers are increasingly committed to establishing early detection and intervention strategies to help alleviate PDAC.

Chronic pancreatitis (CP) is one of the widely recognized risk factors for PDAC. Approximately 5% of patients with CP inevitably develop PDAC [7, 8]. The expression profile of non-coding RNA inevitably changes during the progression of CP to PDAC, resulting in changes in the expression of oncogenes and tumor suppressor genes [9]. A group of non-coding RNA, called long non-coding RNA (lncRNA), regulates mRNA expression through a competitive combination with microRNA (miRNA) [10]. Moreover, lncRNAs are known to play important roles in the progression of normal pancreatic acinar cells into PDAC [11, 12]. The potential carcinogenic effect of lncRNA DLEU1/miR-381/CXCR4 pathway has been reported by comparing the gene expression profiles of PDAC and adjacent normal pancreatic tissues [13]. In addition, lncRNAs play important roles in the proliferation, migration, and invasion of PDAC. The expression of lncRNA *PCAT6* is upregulated in PDAC, while the expression of PCAT6/miR-185-5p/CBX2 axis has been related to TNM stage, lymph node invasion, and overall survival of patients with PDAC [14]. However, the key gene regulatory processes and the function of lncRNAs in the progression from CP to PDAC have not been clarified.

Accumulating evidence shows that mutations and disorders in lncRNAs are related to the development and progress of various complex human diseases. Chen et al. reviewed five important lncRNA-related diseases, five critical disease-related lncRNAs, and some important open lncRNA-related sequences, expression patterns, functions, and other databases. In these articles, it has been pointed out that making full use of different types of heterogeneous data sources can more effectively identify new lncRNA-disease interactions [15, 16]. This account inspired us to focus on the characteristics of carcinogenesis caused by potential differences in lncRNAs between CP and PDAC.

In this study, we identified the different lncRNAs in CP and PDAC. Further, weighted gene co-expression network analysis (WGCNA) was used to screen out gene sets with highly synergistic changes. We created a lncRNA-miRNA-mRNA network to assess the impact and role of lncRNA and mRNA dysregulation in the progress from CP to PDAC. By creating a protein-protein interaction (PPI) network, two key differentially expressed genes (DEGs) and an event module were defined. Finally, quantitative real-time polymerase chain reaction (qPCR) was used to detect the changes in the non-coding RNA and key mRNAs in pancreatic tissues collected from patients with CP and PDAC.

**Fig. 1:**
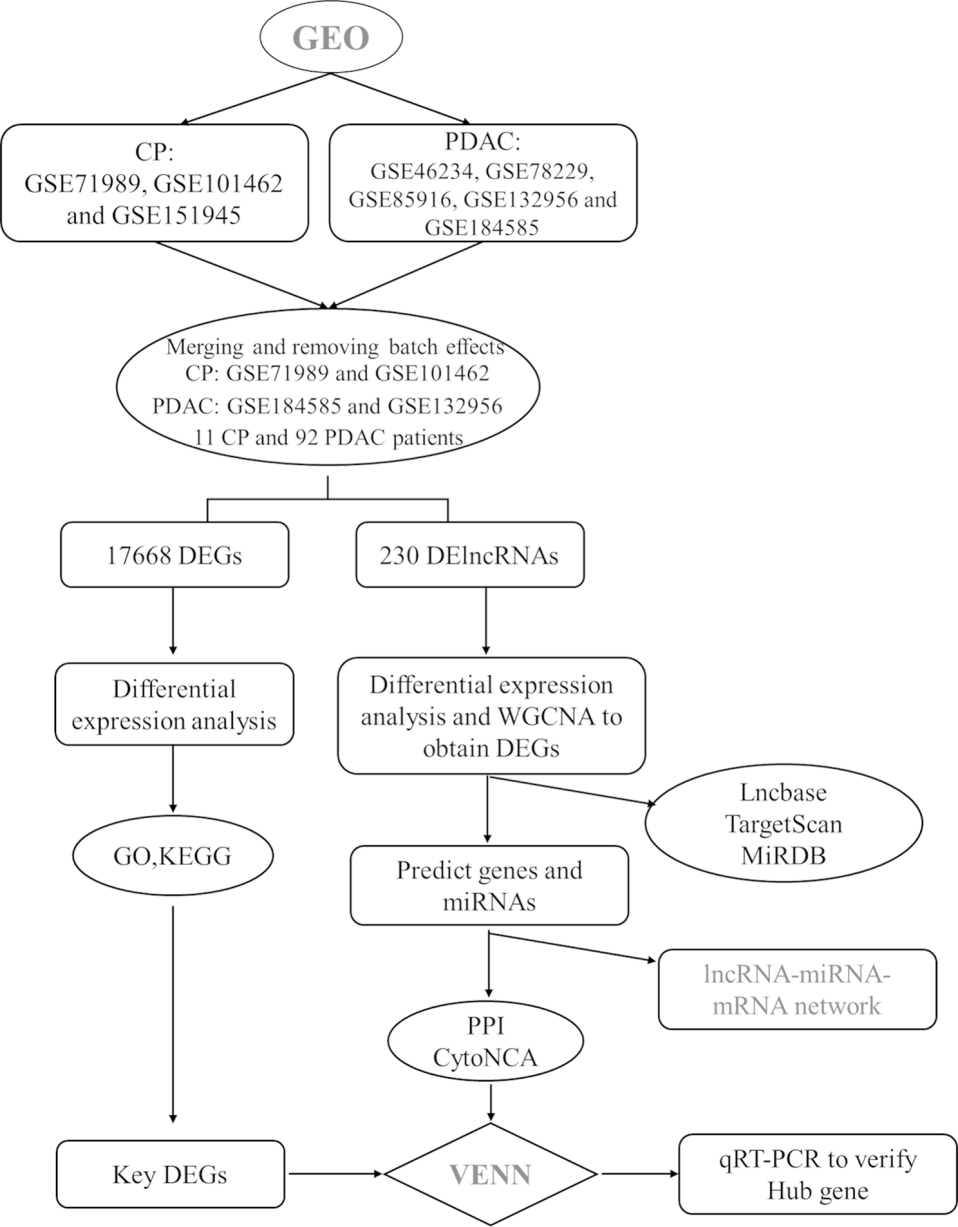
Overall design of the study

## Results

### Data acquisition and normalization

**Fig. 2:**
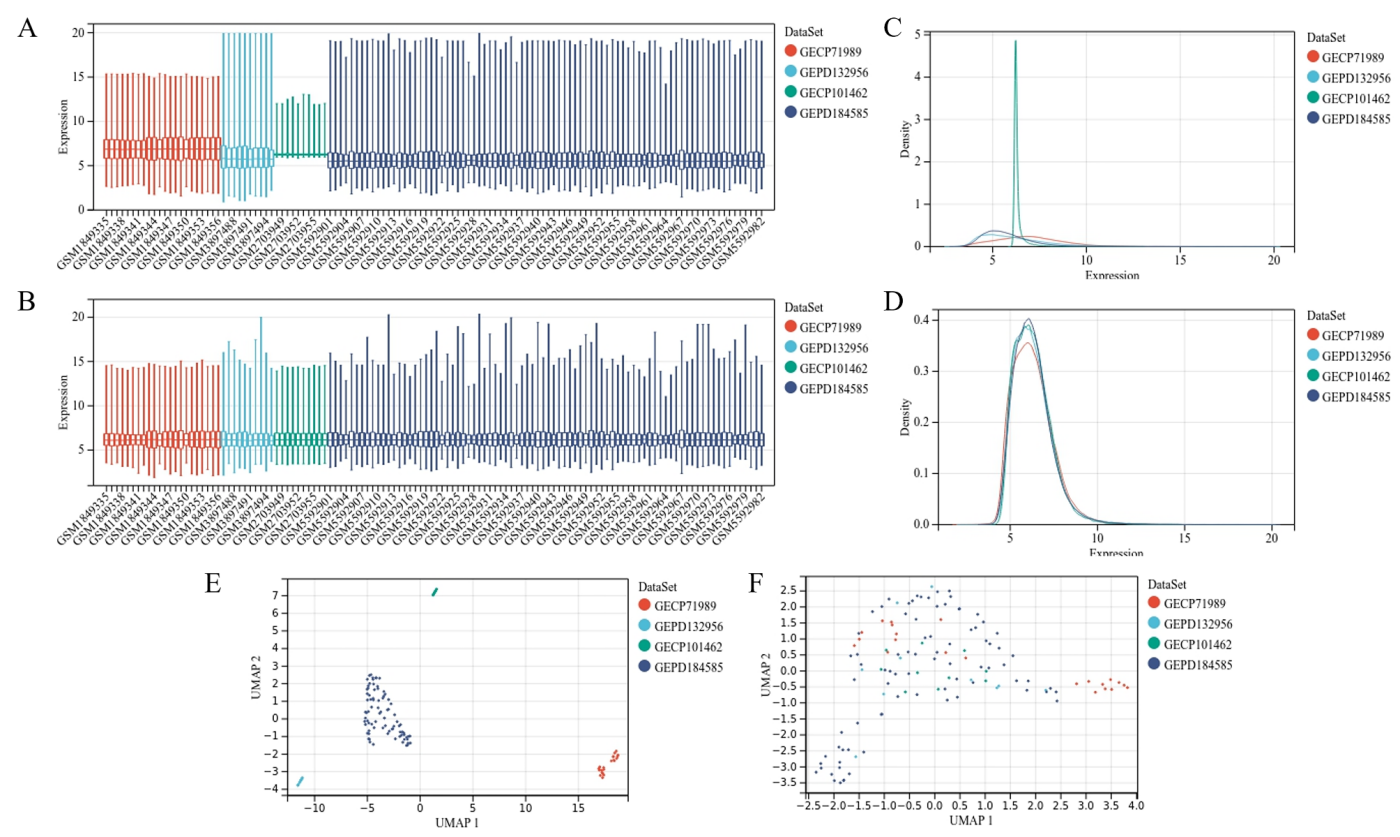
**Dataset merging and consistency testing. Boxplots: (a)** The sample distributions of individual datasets before the removal of the batch effect are illustrated. **(b)** Consistent distribution of data among individual datasets after the removal of the batch effect is presented with the median value on one line. **Density chart: (c)** Sample distribution of each dataset before the removal of the batch effect is presented. **(d)** The consistent distribution between each dataset after the removal of the batch effect is illustrated using the mean and variance close. **UMAP graphs: (e)** Clustering of the samples from each dataset before the removal of the batch effect is depicted. **(f)** The clustering of the samples from each dataset after the removal of the batch effect is shown

In the present study, intersection analysis of 2 PDAC and 2 CP datasets was included. A boxplot was used to visualize the distribution of data among individual datasets, which was consistent after removing the batch effect (Fig. 2a and b). Subsequently, the density and UMAP maps suggested that the samples among the datasets were clustered together (Fig. 2c, d and e, and 2f). Finally, 230 lncRNAs and 17,668 mRNAs were included in this study.

### Volcanic and thermal maps and enrichment analysis of DEGs and DElncRNAs

**Fig. 3:**
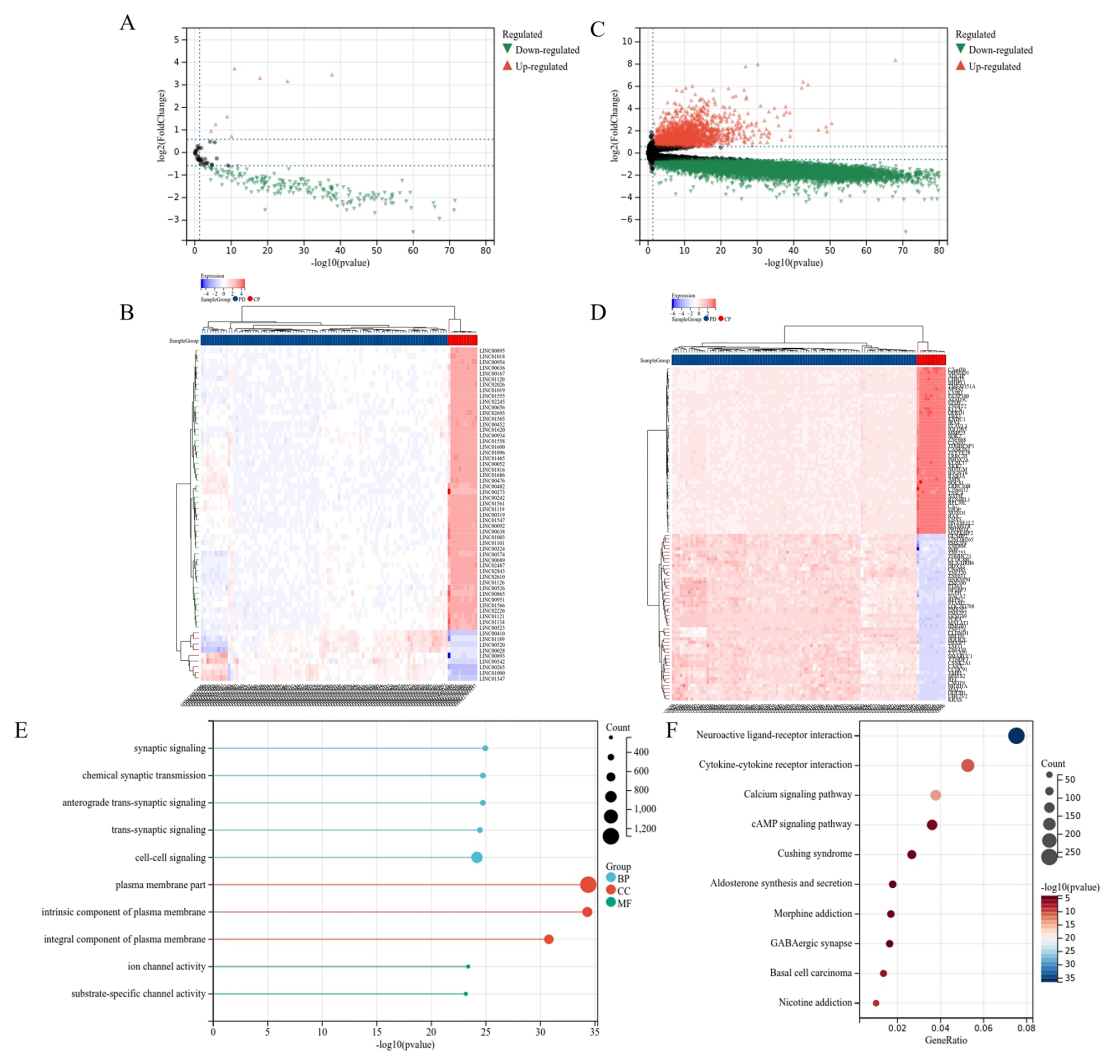
**Differentially expressed genes and enrichment analysis. (a, c)**: Volcano plots depicting the differentially expressed long non-coding RNAs (lncRNAs) or mRNAs with |log2FC|>1.5 and FDR < 0.05 is illustrated. The red and green dots represent the upregulated and downregulated genes, respectively. **(b, d)** The DElncRNAs and DEGs with the least FDR among upregulated and downregulated genes were considered, respectively, for heatmap building. **(e, f)** The three main GO terms and KEGG pathways of the DEGs are shown

PDAC and PC gene expression matrices were used as the experimental and control groups, respectively, to establish differential analysis. *P*-adjust value < 0.05 was selected as the standard of significance for difference analysis. The results revealed nine upregulated lncRNAs and 188 downregulated lncRNAs (Fig. 3a and b). In addition, 2334 upregulated differential mRNAs and 10,341 downregulated differential mRNAs were included in the enrichment analysis (Fig. 3c and d).

The gene ontology (GO) enrichment results indicated the potential involvement of differentially expressed mRNAs (DEGs) in biological processes (BPs), cellular components (CCs), and molecular functions (MFs) (Fig. 3e). Specifically, our GO enrichment results revealed the involvement of chemical synchronous transmission, cell signaling, and ion channel activity. Analysis of the Kyoto Encyclopedia of Genes and Genomes (KEGG) enrichment results identified major biological pathways including cytokine–-cytokine receptor interaction, calcium signaling pathway, cAMP signaling pathway, and nicotine addiction, which were significantly different (Fig. 3f).

### WGCNA and a potential lncRNA-miRNA-mRNA regulatory network

**Fig. 4:**
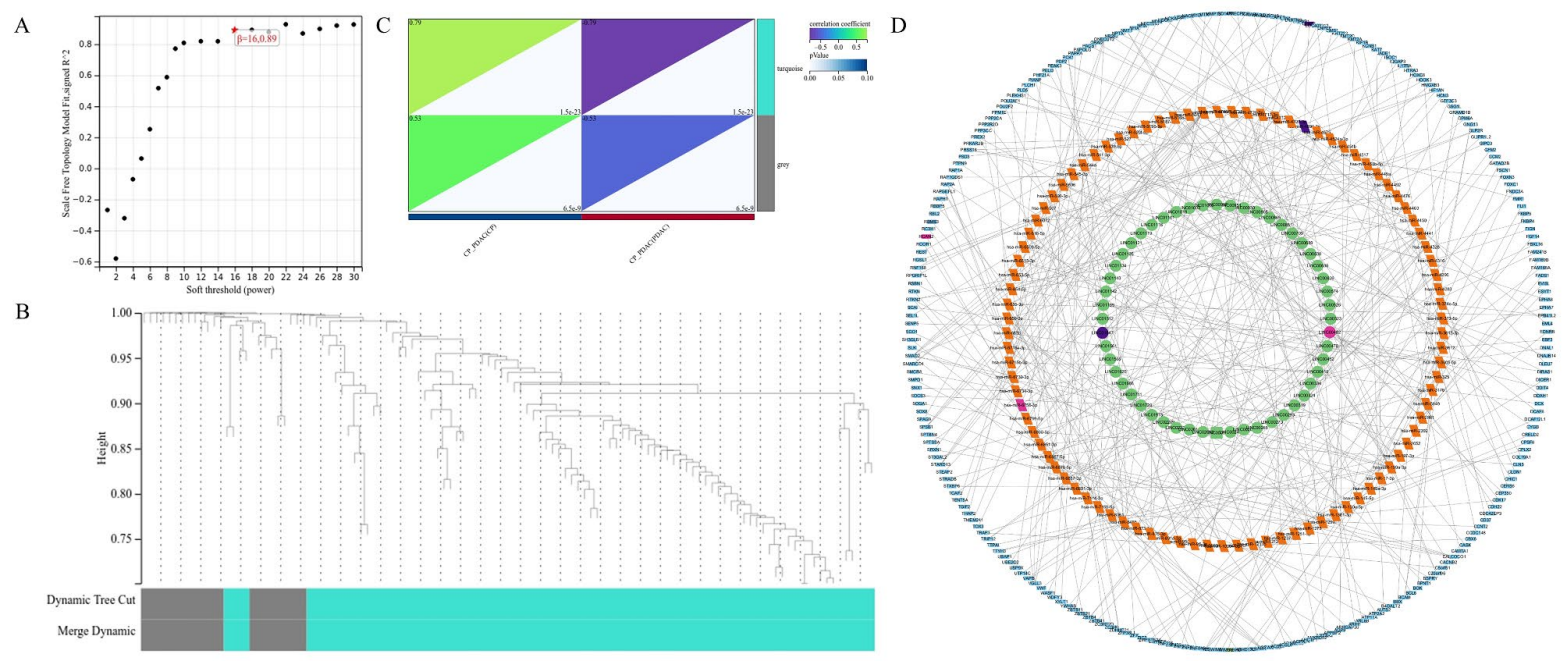
The gene modules associated with chronic pancreatitis (CP) and pancreatic ductal adenocarcinoma (PDAC), as identified using weighted gene co-expression network analysis, are depicted here. **(a)** Interconnections among the network genes show scale-free network distribution at a soft threshold power (β) of 16 (scale-free R^2^ = 0.89). **(b)** The dendrogram indicates the classification of genes into distinct modules according to correlation analysis. Each color represents a module, and the gray module includes genes that are not assigned to a particular network. **(c)** The heatmap of module trait relationships shows the association of various gene modules with the clinical status. **(d)** The potential lncRNA–miRNA–mRNA regulatory network is shown

We constructed a gene co-expression network of all DElncRNAs and screened 52 key regulatory lncRNAs using WGCNA. LncRNA plays a key role in regulating mRNA expression through endogenous competition and miRNA (Fig. 4a and b, and 4c). Hence, the miRNAs and genes regulated by lncRNA were included in this study (Table S1). A total of 52 lncRNAs, 104 miRNAs, and 312 mRNAs were included to construct a potential lncRNA-miRNA-mRNA regulatory network (Fig. 4d).

### PPI network and identification of hub genes

**Fig. 5:**
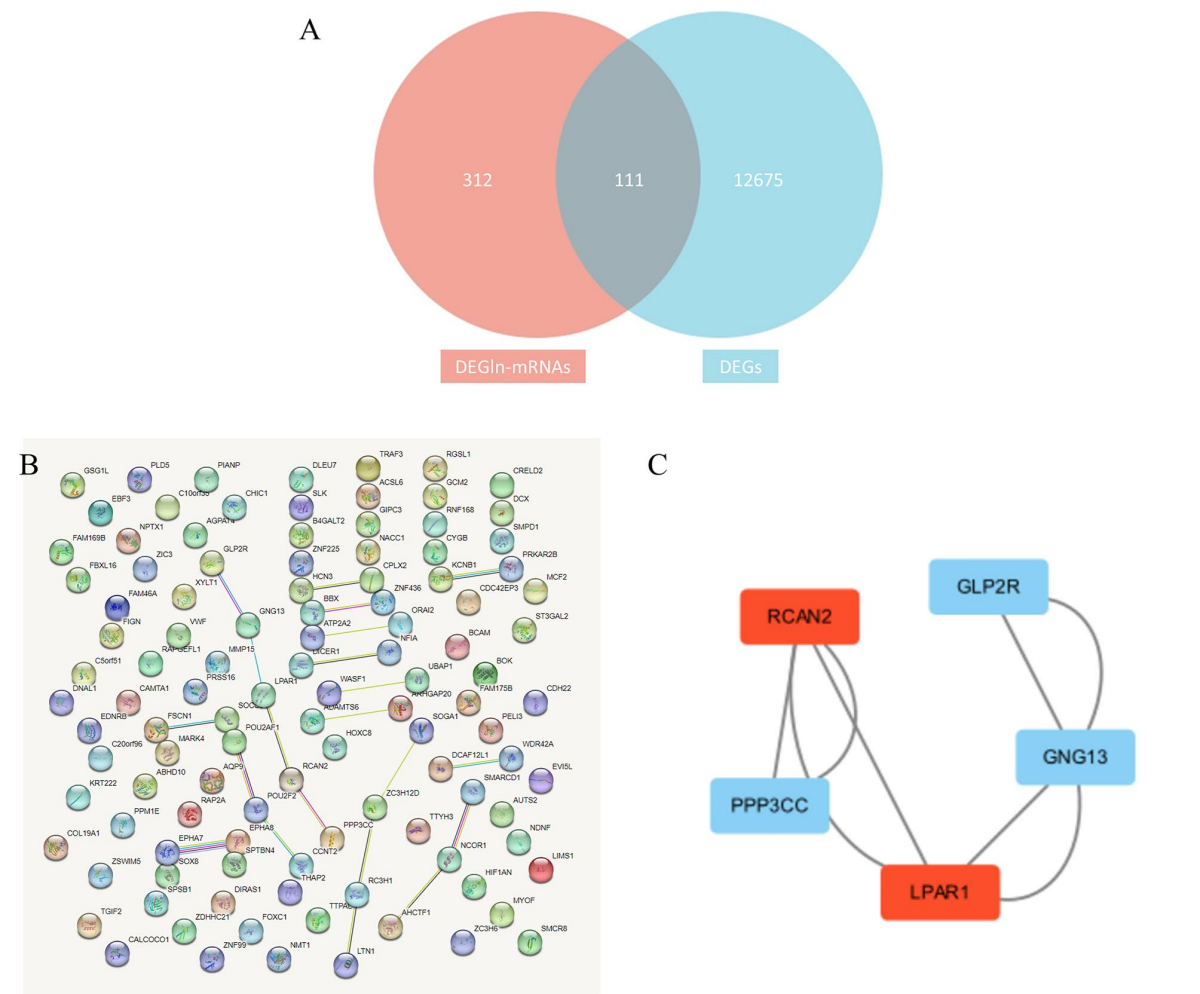
**(a)** Venn diagram denoting the 111 DElncRNAs regulated by DEGs obtained by determining the intersection of the mRNAs regulated by DElncRNAs and DEGs, is shown here. **(b)** Protein-protein interaction network of 111 DElncRNAs regulated by DEGs is illustrated. **(c)** The core network of protein with the largest bridging centrality (BC) value in the PPI network is depicted

After determining the intersection of the mRNAs regulated by DElncRNAs and DEGs, a total of 111 DElncRNAs regulated by DEGs were obtained. The PPI network was established using 111 DEGs. In cytoHubba, the core gene groups (Fig. 5a), including lysophosphatidic acid receptor 1 (*LPAR1*), protein phosphatase 3 catalytic subunit gamma (*PPP3CC*), a regulator of calcineurin 2 (*RCAN2*), glucagon-like peptide 2 receptor (*GLP2R*), and G protein subunit gamma 13 (*GNG13*), were screened out according to the MCC algorithm. Furthermore, the results revealed that two of the five central DEGs of the PPI network were created in this module, suggesting that *LPAR1* and *RCAN2* might have an important role in the progression from CP to PDAC (Fig. 5b and c).

### Validation of hub genes using qPCR

**Fig. 6:**
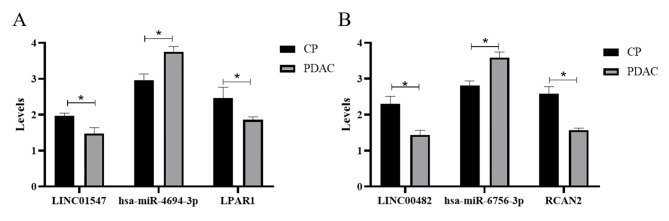
The results of quantitative polymerase chain reaction (qPCR) analysis to elucidate mRNA expression levels are shown. **(a)** The expression levels of *LINC01547* and *LPAR1* in chronic pancreatitis (CP) are significantly higher than those in pancreatic ductal adenocarcinoma (PDAC); expression levels of hsa-miR-4694-3p in PDAC are significantly higher than those in CP (*P < 0.05). **(b)** Expression levels of LINC00482 and RCAN2 in CP are significantly higher than those in PDAC; expression levels of hsa-miR-6756-3p in PDAC are significantly higher than those in CP (*P < 0.05)

qPCR results revealed that compared to the CP group, the expression of *LINC01547* and *LPAR1* in the PDAC group reduced (*P* < 0.05). By contrast, the expression of hsa-miR-4694-3p was increased in the PDAC group (*P* < 0.05). Similar results were obtained for the LINC00482/hsa-miR-6756-3p/RCAN2 (Fig. 6, *P* < 0.05,). In addition, the demographic and clinical characteristics of six patients were shown in Table 1.

**Table 1:** Demographic and clinical characteristics

Patients	Diagnoses	Age	Gender	Height (cm)	Weight (kg)	HBP	DM	ASA	Hb (g/dL)	CRP (mg/L)	CA199 (U/mL)
A	PDAC	70	Male	158	62	N	+	2	14.0	49.5	164.8
B	PDAC	62	Female	155	40	N	N	1	12.3	82.8	20.3
C	PDAC	53	Male	160	55	+	N	1	15.8	121.8	10.6
D	CP	46	Female	153	47	N	N	1	12.6	22.3	12.8
E	CP	47	Male	165	64	N	N	1	13.7	19.3	13.9
F	CP	41	Female	155	61	N	N	1	13.4	51.0	5.2

PDAC, pancreatic ductal adenocarcinoma; CP, chronic pancreatitis; HBP, hypertension; DM, diabetes mellitus; ASA, American Society of Anesthesiologists; Hb, hemoglobin; CRP, C-reactive protein; CA199: Carbohydrate antigen 199.

## Discussion

*LPAR1* and *RCAN2* were considered as the hub genes, based on the PPI network analysis. After further investigation, we found that the non-coding RNA LINC01547/hsa-miR-4694 was regulated upstream of *LPAR1*, while LINC00482/hsa-miR-6756-3p was regulated upstream of *RCAN2*. Hence, we observed two independent signal transduction systems in the progression of CP to PDAC. *LINC01547* on chromosome 21 can promote the expression of *LPAR1* by combining with hsa-miR-4694-3p, playing a role in inhibiting the development of PDAC. In addition, our study found that *LINC00482* on chromosome 17 played an important role in the development of PDAC. LINC00482 promotes *MMP15* expression by recruiting FOXA1, leading to the inhibition of inflammation and angiogenesis in bladder cancer [17], consistent with our results. Low expression of *LINC00482* (*P* < 0.05) led to the enrichment of hsa-miR-6756-3p, which suppressed *RCAN2* expression. Although CP and PDAC are often clinically indistinguishable, the low expression of two classes of lncRNAs identified in pancreatic tissues of patients with CP may have potential diagnostic value [18].

Our results suggest that *LPAR1* and *RCAN2* play important roles in the progression of CP to PDAC. The first key mRNA between CP and PDAC identified was *LPAR1*. Lysophosphatidic acid (LPA) has been proven as a driver of melanoma cell chemotaxis and invasion [19]. In this investigation, excessive LPA signaling led to collagen remodeling, and *LPAR1* was a potentially important target in this process. Multiple pathways, including angiogenesis, are controlled by LPAR1 signaling. LPA signaling through LPAR1 contributes to the inhibition of angiogenic processes in cancer cells [20]. Further analysis of the non-coding RNAs regulated upstream of *LPAR1* identified the regulatory pathway of the LINC01547/hsa-miR-4694-3p/LPAR1 signaling axis. The second key differential mRNA identified in our study was RCAN2. As an inhibitor of calcineurin, RCAN2 regulates calcineurin nuclear factor (NFAT) signal transduction of activated T cells, resulting in altered differentiation/proliferation, metabolism, and oxidative stress response [21, 22]. As a result, PDAC initiation may rely on NUAK2-RCAN2 dysregulation triggered by persistent expression of mutant p53. The structural lesion or dysfunction of the *p53* gene is one of the most common genetic events in cancer. Compared with the wild cohort, the expression of *NUAK2* and *RCAN2* in the mutant p53 cohort was downregulated [23]. A study identified *NUAK2* and *RCAN2* as differentially regulated genes with concordant prognostic value in human PDAC harboring *p53* mutations, suggesting that *NUAK2* and *RCAN2* are novel candidate genes involved in tumor-promoting networks induced by *p53* mutations [24]. Similarly, we tracked the regulatory pathway of the LINC00482/ hsa-miR-6756-3p/RCAN2 signaling axis. Further study of lncRNA-protein interactions (LPIs) may facilitate the understanding of the biological functions and mechanisms of LINC01547 and LINC00482. Compared to the wet experiment, using the calculation method to reveal the possible correlation is faster and cheaper. The depth model including deep neural networks and deep forests can effectively learn the representative characteristics of lncRNAs and proteins [25, 26].

PDAC is a digestive system tumor with high malignancy, rapid disease progression, and extremely poor prognosis [27]. Researchers currently pay more attention to devising effective treatment strategies to alleviate PDAC, while the role of key genes, especially lncRNAs, in the progression of CP to PDAC remains elusive [28, 29]. LncRNAs belong to a class of competing endogenous RNAs (ceRNAs) and act as molecular microRNA (miRNA) sponges that control mRNA expression by adsorbing miRNAs as miRNA response elements (MRE) [30]. LncRNAs exhibit greater potential as diagnostic markers and therapeutic targets than traditional mRNAs and proteins due to the following unique advantages. LncRNA is more representative of disease features because its expression varies widely in different tissues, diseases, and stages of disease progression. Moreover, lncRNAs can directly participate in various biological processes, and the dynamic changes in their expression are closely related to the characteristics of diseases.

LncRNAs have been identified as key regulators of the pathogenesis and progression of PDAC and are considered as promising biomarkers of this type of cancer [31]. Macrophages isolated from the tumor core of PDAC were subsequently used to construct a stable tumor cell line with lncRNA-PACER knockdown and inoculated subcutaneously into nude mice to confirm their cancer-promoting function [32]. This study revealed that LncRNA-PACER sponges miR-671-3p to activate the KLF12/AKT/c-myc pathway and interacts with insulin-like growth factor 2 mRNA-binding protein 2 (IGF2BP2) to enhance the stability of KLF12 and c-myc. Previous studies evaluating lncRNA FLVCR1-AS1 reported that overexpression of lncRNA FLVCR1-AS1 significantly inhibited PC cell proliferation, cell cycle, and migration in vitro and in vivo [33]. Notably, several lncRNAs have been shown to play important roles in PDAC chemoresistance to gemcitabine, including lncRNA HOTTIP and GAS5 [34]. In summary, lncRNA can regulate the growth, invasion, migration, and angiogenesis in the tumor microenvironment as well as gemcitabine resistance in the development of PDAC [35]. Our PCR results revealed a significant difference in the expression of *LINC01547* and *LINC00482* between patients with CP and patients with PDAC, suggesting that these two non-coding RNAs might play an important role in the progression of CP to PDAC by influencing different mRNAs. Relevant studies have also suggested that lncRNAs, in plasma exosomes and pancreatic tissue of patients, have potential applications in the differential diagnosis of CP and PDAC [36, 37].

Further exploration of our study results is possible; our study has a few limitations. First, we demonstrated that low expression of *LINC01547* and *LINC00482* in the PDAC group, compared with the CP group, could indicate the potential of these lncRNAs as diagnostic markers. However, the expression of these lncRNAs in the blood of the two groups was not examined due to sample limitation. Second, although we found that *LPAR1* and *RCAN2* were potential therapeutic targets, in vivo and in vitro experiments were not performed to confirm and validate the findings. Finally, future multicenter studies involving large sample sizes will help support the conclusions of this study.

## Conclusion

Two signaling axes critical in the progression from CP to PDAC were screened out by merging multiple CP with PDAC datasets. Results of qPCR experiments suggested that LINC01547/hsa-miR-4694-3p/LPAR1 and LINC00482/ hsa-miR-6756-3p/RCAN2 might play important roles in the progression of CP to PDAC. These findings provided novel insights into the molecular mechanism and potential diagnostic or therapeutic biomarkers for CP and PDAC. Future studies could explore the differential diagnostic value of lncRNAs and validate the roles of downstream mRNAs in cancer therapy.

## Methods

### Microarray data and organization source

In this study, the keywords “chronic pancreatitis” or “pancreatic ductal adenocarcinoma” were used to obtain microarray data from the Gene Expression Omnibus (GEO) database (https://www.ncbi.nlm.nih.gov/geo/). Five PDAC (GSE46234, GSE78229, GSE85916, GSE132956, and GSE184585) and three CP datasets (GSE71989, GSE101462, and GSE151945) were included in this study. The matrix files were annotated with an official gene symbol utilizing the microarray platform’s data table, generating gene expression matrix files, which were combined into a single file. Subsequently, the “limma” R package was used to remove the batch effect and eliminate the sample sets with excessive differences (R software package inSilicoMerging) [38]. The datasets whose intersection was less than 5% were excluded in this step. Finally, two CP (GSE71989 and GSE101462) and two PDAC datasets (GSE184585 and GSE132956) were included in the final analysis. The merged dataset included 103 samples, including data collected from the pancreatic tissues sourced from 11 patients with CP and 92 patients with PDAC, respectively. The overall design of the study is shown in Fig. 1.

To further elucidate the difference in lncRNA expression in CP and PDAC, we collected samples from patients undergoing surgery for CP and PDAC in our department and detected the differential non-coding RNAs and mRNAs screened using PCR. A total of 182 consecutive case reports were collected from our institution. These case reports included patients who underwent pancreaticoduodenectomy between 2018 and 2021. Twenty-five patients were excluded based on various criteria, including an American Society of Anesthesiologists (ASA) grade of above 3, previous history of diagnosis of malignant tumors, combined with a history of organ resection (apart from the pancreas), and insufficient clinical data. Finally, pancreatic tissues collected from 41 patients with PDAC and 3 patients with CP were stored at − 80 ° C in the pathology room of our department. Considering the statistical significance and the matching of sample size between the two groups, we selected three pancreatic tissues collected from each of the PDAC and CP groups for PCR detection. The General Hospital of Western Theater Command Ethics Committee has approved the study, and all the procedures follow the principle of no harm to the human body (2021EC2-26).

### Differential expression analysis

The original data were normalized and logarithmically converted. The Bioconductor Limma package was used for defining differentially expressing lncRNAs (DElncRNAs) and mRNAs (DEGs) in each dataset. Subsequently, we screened differential expressed mRNAs and lncRNA according to the standard criteria of log2 fold change (FC) > 1.5 and *P*-adjust value < 0.05 (False Discovery Rate, FDR).

### GO functional analysis and KEGG pathway

GO analysis was performed for assessing the potential involvement of DEGs and DElncRNAs in biological process (BP), cell composition (CC), and molecular function (MF). Additionally, KEGG analysis was performed to identify major biological functions and pathways involving the DEGs [39, 40]. GO and KEGG analyses were performed using the R package clusterProfiler (version 3.14.3). The ggplot2 software package in R studio and R scripting language was used for visualizing the analysis.

### WGCNA and prediction of non-coding RNA targets

A co-expression network was established using the WGCNA package for screening gene modules with genes showing tight associations (v1.61; https://cran.r-project.org/web/packages/WGCNA/index.html) in R. WGCNA performs diverse functions in weighted correlation network analysis, e.g., network building, module detection, topological property determination, and data simulation, visualization, and interface with the external software. The soft threshold was assessed based on the scale-free topology criterion. The interconnections among the network genes showed a scale-free network distribution at a soft threshold power (β) of 16 (scale-free R^2^ = 0.89). Next, co-expression modules (cut-off height ≥ 0.25) were determined based on a phylogenetic tree. Module analysis provided hierarchical clustering with same-branch modules showing comparable gene expression patterns. Comparable modules were combined into two co-expression modules (nattier blue and grey). Then, gene clusters were visualized, and associations among various modules were assessed. Associations of gene modules with CP and PDAC were examined, wherein modules displaying the most significant positive and negative correlations with clinical characteristics were identified.

We calculated the Medium Absolute Deviation (MAD) for each lncRNA and eliminated the first 50% of the smallest MAD lncRNAs. Furthermore, we used the goodSamplesGenes method of the WGCNA package of the R software to remove the lncRNA outliers. We predicted the target miRNA of the selected lncRNA in LncBase (https://diana.e-ce.uth.gr/lncbasev3/home).

In addition, target mRNAs of miRNA were predicted using miRDB (https://mirdb.org/). Accordingly, we obtained the relationship between lncRNA-miRNA-mRNA and built a ceRNA interaction network diagram using Cytoscape (version 3.9.1) for visualization. Subsequently, mRNAs downstream of DElncRNAs were subjected to intersection analysis with DEGs, and 111 overlapping mRNAs were obtained.

### PPI network and hub genes

Based on the above-intersecting DEGs, the Interaction Gene Retrieval (STRING) database was used to determine the interaction relationship, and the PPI network was constructed using Cytoscape. Then, the cytoNCA plug-in was used to calculate the bridging centrality (BC) values to screen key modules and determine the hub genes.

### Quantitative real-time PCR (qPCR)

Total RNA was isolated and extracted from the specimens. Then, the purified RNA was reverse-transcribed to produce cDNA, and its concentration was determined. The qPCR was performed in a fluorescence quantitative PCR system using the Easy™ Mix SYBR kit. The gene sequences used in this study were listed in Table 2.

**Table 2:** RT-qPCR primer sequences

Genes	Primer sequences
β-actin	F: GGGAAATCGTGCGTGACATT
	R: GCGGCAGTGGCCATCTC
LPAR1	F: GCTGCCATCTCTACTTCCATC
	R: AAGCGGCGGTTGACATAGATT
RCAN2	F: CAGCCCGAGCTAGGATAGAG
	R: CTGGCGTGGCATCGTTGAT
hsa-miR-4694-3p	F: GGGCAAATGGACAGGATA
	R: CAGTGCGTGTCGTGGAGT
hsa-miR-6756-3p	F: GGGUCCCCUUCCUCCCU
	R: CAGTGCGTGTCGTGGAGT
LINC01547	F: AGAGAATCCCTCACCAATAAGGA
	R: AGACCAGTGTTTCCCTCTGTG
LINC00482	F: AGTCCCTTGGGAAGTCACAG
	R: GAACAAGAACAAGGGGGTGG

## Electronic supplementary material

Below is the link to the electronic supplementary material.

**Additional file 1:** **Table S1.** The miRNAs and genes regulated by lncRNA.

## Data Availability

statement. The datasets analyzed during the current study are available in the [GEO] repository, [https://www.ncbi.nlm.nih.gov/geo/ and GSE71989, GSE101462, GSE151945, GSE46234, GSE78229, GSE85916, GSE132956, and GSE184585].
